# Inaugural *BMC Ecology and Evolution* image competition: the winning images

**DOI:** 10.1186/s12862-021-01886-7

**Published:** 2021-08-13

**Authors:** Jennifer L. Harman, Alison L. Cuff, Josef Settele, Luke M. Jacobus, David A. Liberles, Arne Traulsen

**Affiliations:** 1 BMC, London, UK; 2 BMC, Berlin, Germany; 3 Helmholtz-Centre for Environmental Research – UFZ, Leipzig, Germany; 4 Indiana University-Purdue University Columbus (IUPUC), Columbus, IN USA; 5 Temple University, Philadelphia, PA USA; 6 Max-Planck Institute for Evolutionary Biology, Schleswig-Holstein, Germany

## Abstract

The inaugural *BMC Ecology and Evolution* image competition attracted entries from talented ecologists and evolutionary biologists worldwide. Together, these photos beautifully capture biodiversity, how it arose and why we should conserve it. This editorial celebrates the winning images as selected by the Editor of *BMC Ecology and Evolution* and senior members of the journal’s editorial board.

In celebration of the launch of *BMC Ecology and Evolution*, we are delighted to announce the winning images of the 2021 photography competition. *BMC Ecology*, which merged with *BMC Evolutionary Biology* this year to create *BMC Ecology and Evolution,* has run the competition for several years [1–7]. In common with previous years, the 2021 competition has produced an impressive collection of images and we would like to congratulate all our winners. The competition attracted entries from researchers all around the world eager to use their creativity to highlight their work and capture the diversity of the planet's flora and fauna. *BMC Ecology and Evolution* invited anyone affiliated with a research institution to submit to one of the following six categories: ‘Conservation Biology', 'Evolutionary Developmental Biology and Biodiversity', 'Behavioural Ecology', 'Human Evolution and Ecology', ‘Population Ecology' and 'Ecological Developmental Biology'.

Our Senior Editorial Board Members lent their expertise to judge the entrants to the competition, selecting the overall winner, runner up and best image from each category. The board members considered the scientific story behind the photos submitted in addition to their artistic judgement (Fig. 1).

## Overall winner and best image for ‘Conservation Biology’

**Fig. 1:**
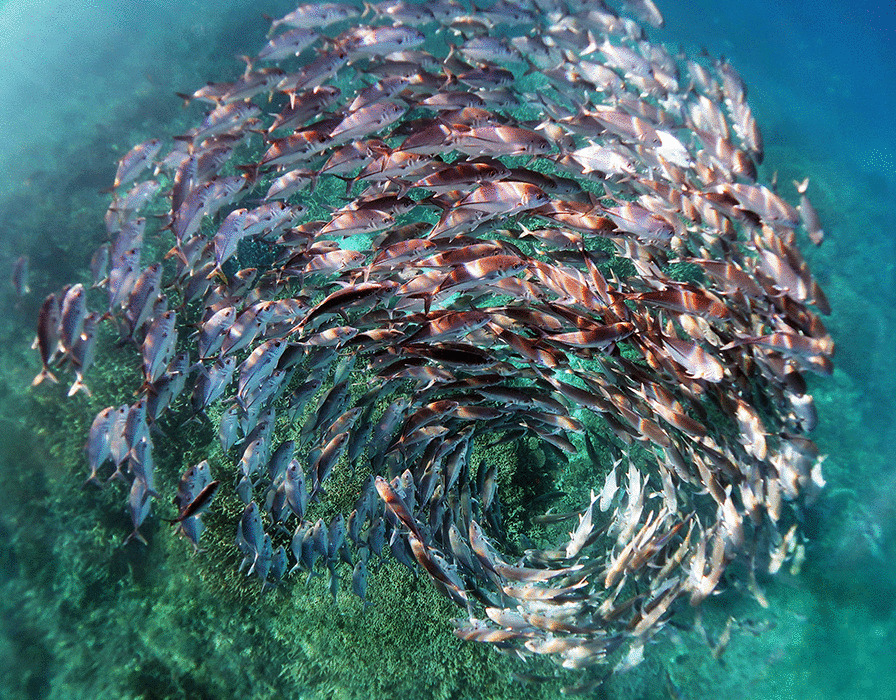
A school of jack fish in a spiral formation at Heron Island in the Great Barrier Reef. A visual metaphor for the spiralling crisis unfolding within our oceans and the need for concentrated efforts to protect marine ecosystems. Attribution: Kristen Brown

Marine biodiversity sustains life and the health of our planet. However, human activities are threatening the well-being of the world’s oceans. Global warming, pollution and overfishing are having a detrimental impact on the functioning of marine ecosystems, including the world’s coral reefs. Kristen Brown, a coral reef ecologist, submitted our overall winning image, which captures a school of jack fish in a spiral formation. Kristen describes the image as a visual metaphor for the “spiralling crisis unfolding” within our oceans. Kristen eloquently explains that “This image represents both the beauty and bounty of our oceans as well as the spiralling crisis unfolding within the marine environment. Coral reefs with high coral cover and plentiful fish populations like this one at Heron Island on the Great Barrier Reef are sadly becoming rarer. Without a concentrated effort to reduce greenhouse gas emissions and improve water quality, coral reefs as we know them are at risk of disappearing within our lifetime.” Senior Editorial Board Member Josef Settele says that the image is “also a symbol for the need for concentrated efforts to manage biodiversity loss and set conservation priorities”. Kristen’s research focuses on the impact of global change on coral reef ecosystems. Climate change is causing alterations in ocean chemistry, leading to ocean warming and acidification. As a postdoctoral researcher at the University of Pennsylvania, Kristen works with Assistant Professor Katie Barott to research acidification stress in corals. Kristen is also an associate research fellow at the University of Queensland in Australia investigating the potential trade-offs or compromises made by corals within the Great Barrier Reef that have survived recent thermal stress events (Fig. 1).

## Runner up and best image for ‘Evolutionary Developmental Biology and Biodiversity’

**Fig. 2:**
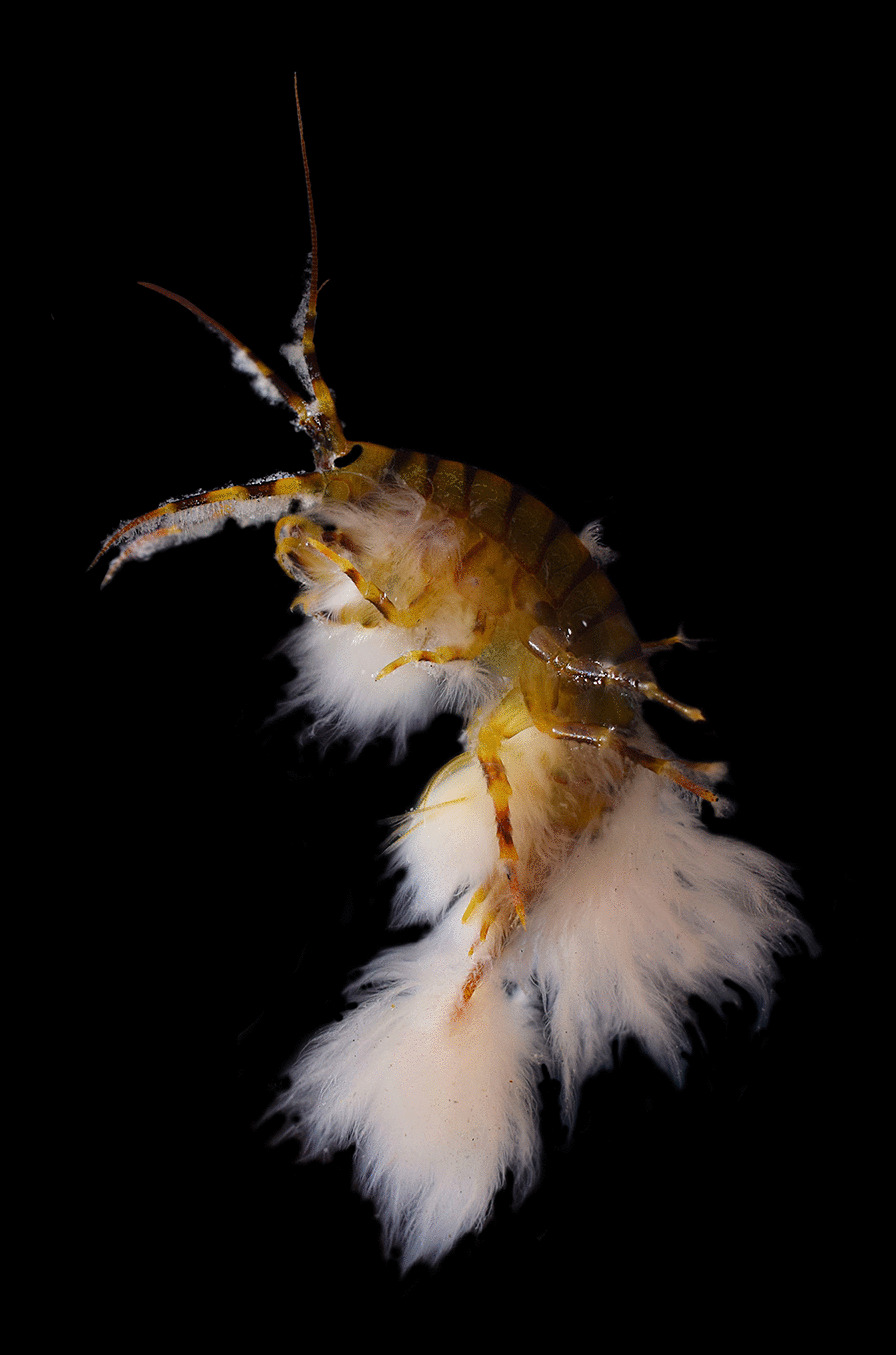
*Eulimnogammarus verrucosus*, a species of crustacean endemic to the UNESCO World Heritage Site Lake Baikal, suffering from a parasitic ciliate and unknown oomycete (water mold) or fungi infection. Attribution: Kseniya Vereshchagina

Kseniya Vereshchagina, an ecologist who studies Lake Baikal in south-eastern Siberia, is our runner up and winner for this category. Kseniya and her lab group at Irkutsk State University in Russia research a species of deep-water crustacean only found within Lake Baikal. Senior Board Member David Liberles comments that the image depicts “a kind of biology usually not visible”. Lake Baikal is one of the oldest and deepest lakes in the world, giving rise to rich and particularly unique freshwater fauna. However, despite being listed as a UNESCO World Heritage Site, Lake Baikal is experiencing an ecological crisis. Kseniya explains that in areas of heavy industry and active tourism, "there is a significant impact on coastal communities." Nutrient runoff from land-based human activities leads to "the restructuring of local ecosystems". Kseniya and her lab group at Irkutsk State University found that in areas of intense human activity, the immunity of endemic amphipod crustaceans is weakened, making them more susceptible to parasitic infection. This image shows "an amphipod crustacean of the species* E. verrucosus* densely covered with an overgrown colony of parasitic ciliates and unknown oomycetes or fungi. These organisms on weakened crustaceans are capable of forming vast colonies resembling a "fur coat". Unfortunately, the crustaceans dressed in such a "fur coat" are sentenced, since the ciliates parasitizing them lead endemics to rapid death." Lake Baikal holds exceptional scientific value to ecologists and evolutionary biologists. Such research highlights the need to minimise the impact of human activity on this precious site (Fig. 2).

## Behavioural ecology

**Fig. 3:**
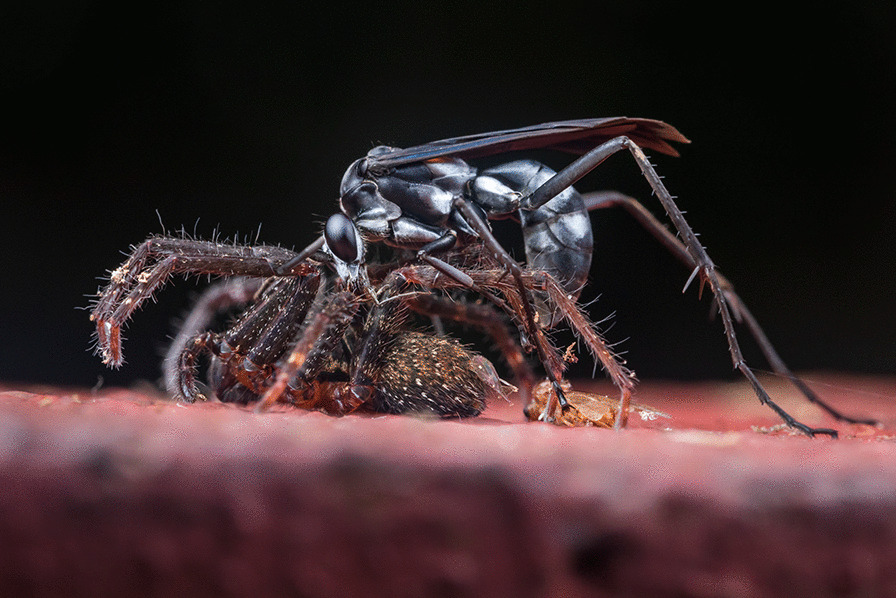
‘The Hunter’ depicts a wasp and its spider prey in Tiputini, Ecuador. Attribution: Roberto García-Roa

The winning image in this category is of a spider that a wasp has captured. This stark image was submitted by Roberto García-Roa, an evolutionary biologist and conservation photographer affiliated with the University of Valencia, Spain. His interests cover several research fields, including ecology, evolution, animal behaviour, animal communication and physiology. Roberto explains that he uses photography “to not only offer an artistic view of what I am living, and from what I am learning, but also to create an attractive and strong link between society and nature. For this purpose, I combine the power of images with the story and / or scientific knowledge behind them to open new avenues of understanding between our culture, as human beings, and our role as a key species to maintain and protect our planet.” Roberto tells us that “Spiders are one of the most sophisticated hunters on earth. Nevertheless, they cannot escape from what evolution has provided to other species. In particular, some groups of wasps are specialized in hunting spiders and use them as a trophic resource for their larvae. I found this epic scene in a wall of a biological station in Tiputini, Ecuador” (Fig. 3).

## Population ecology

**Fig. 4:**
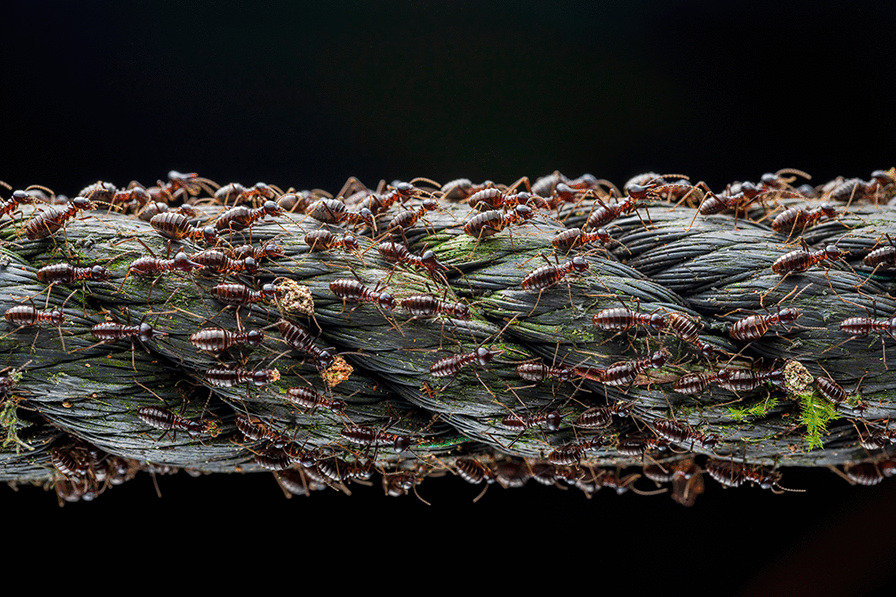
‘Small Big Migration’ captures a moment in the life of a population of soldier termites as they migrate to ensure survivorship and reproduction of the colony. Attribution: Roberto García-Roa

An image captured by Roberto García-Roa was also selected as the best image for the "Population Ecology" category. Luke Jacobus states that "this image clearly illustrates a moment in the life of a population". Roberto writes, "Not all big migrations occur in Africa. Thousands of soldier termites (*Longipeditermes longipes*) are able to migrate in a complex social environment where each individual has its own mission framed altogether in a global objective: the survivorship and reproduction of the colony. In this case, these termites used meters of an abandoned rope to move across the Malaysian forest. Once humans disappear, nature recovers its space and uses what is needed to survive” (Fig. 4).

## Human evolution and ecology

**Fig. 5:**
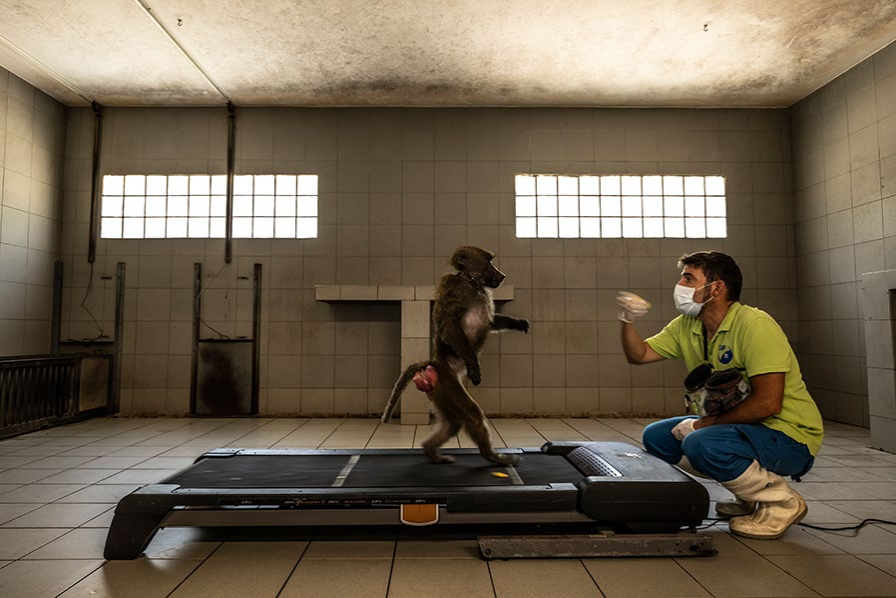
“Learning to Be Human” captures a researcher using a baboon to study the evolution of human locomotion. Attribution: Roberto García-Roa

Our winner in this category entitled “Learning to Be Human” was captured by Roberto García-Roa. Primates can be useful models to study the evolution of human locomotion. To caption this winning image, Roberto García-Roa writes, “To understand our present and predict our future, humans aim to gain enough knowledge to fill the gap of our past. Bipedalism, for example, is probably one of the most critical steps in our evolutionary history. How did it happen? With just a few seconds to capture this scene within the Station of Primatology in France, I was allowed to photograph how a species of primate called *Papion olivaceo* learnt to walk on two legs in a project that aims to investigate the evolution of bipedalism” (Fig. 5).

## Ecological developmental biology

**Fig. 6:**
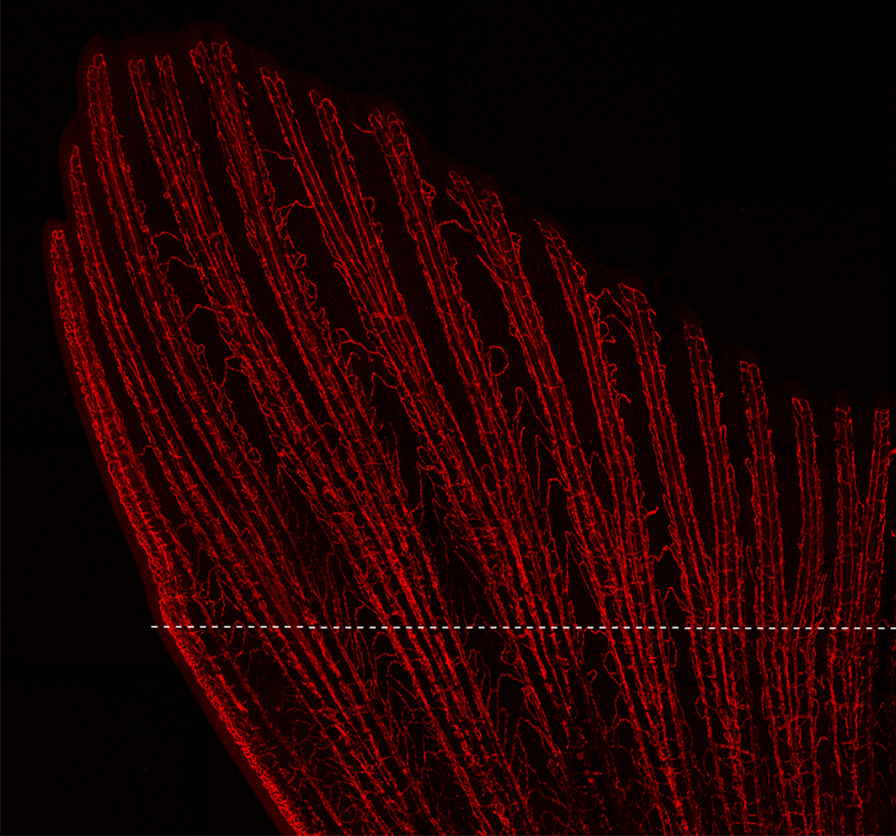
A zebrafish regrew its tail fin only two weeks after the appendage was clipped at the white horizontal dotted line. Attribution: Chey Chapman

Our winner in this category was an entry by Chey Chapman, a PhD student studying the mechanisms underlying zebrafish tissue regeneration at the Royal Veterinary College, the University of London. Mammals cannot repair severe damage to tissues—a severed limb does not grow back. However, many phylogenetically primitive vertebrates, such as zebrafish have a spectacular ability to regenerate various tissues after traumatic injury. Chey tells us that "This image shows the blood vessels in a regenerated zebrafish tail fin. The cells forming the blood vessels are labelled with a red fluorescent reporter. Understanding the mechanisms underlying tissue regeneration is a fascinating challenge. Regenerative capacity can vary greatly among species, and by studying organisms capable of regeneration, we can provide insights to develop strategies for potential therapeutic benefit. Whether regeneration is a primitive or adaptive trait to environmental conditions is the subject of much debate, and the mechanisms underlying the regeneration process are not yet fully understood. Transgenic zebrafish, such as the line used to generate this image, are an important tool to help us better understand why some animals have the power of regeneration by allowing the visualisation of certain cell types labelled by fluorescent reporters" (Fig. 6).

## Editor’s pick

**Fig. 7:**
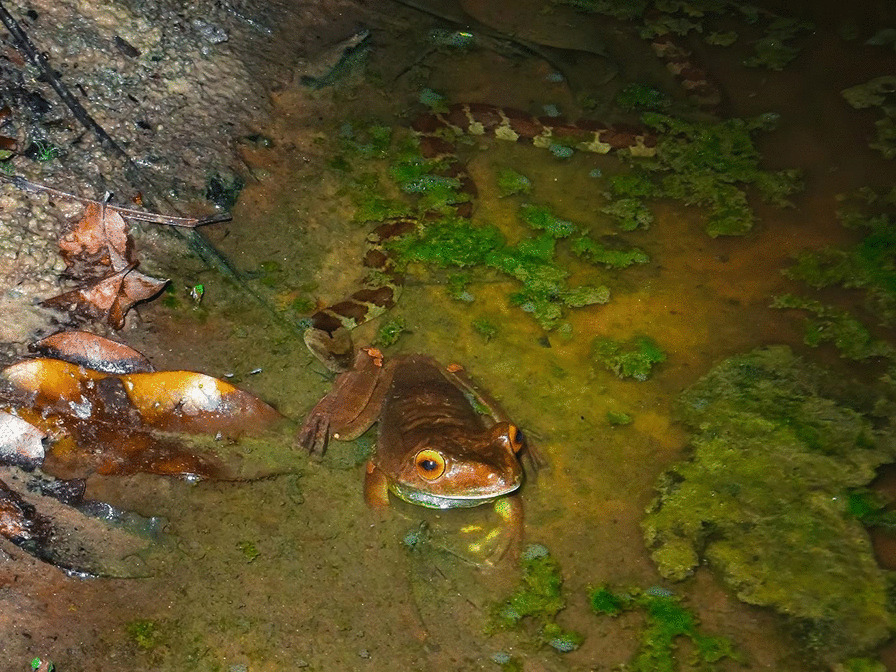
“Eerie Stalker” depicts a giant gladiator frog’s escape from a snake. Attribution: Dimitri Ouboter

My choice as the Editor, which captures a giant gladiator frog's escape from a snake, was submitted to the 'Behavioural Ecology' category. The image was taken by Dimitri Ouboter, an ecologist from the Institute for Neotropical Wildlife and Environmental Studies. The institute is in Suriname, a highly biodiverse country located in South America, almost completely covered by pristine tropical rainforest. Dimitri says, "It isn't often you come upon the behavioural interaction of two species so I was ecstatic to be able to capture part of it. I was walking at night surveying some pools when I saw this snake sneak up on a frog. Seconds after this photo was taken, the snake (*Helicops angulatus*) struck out at this giant gladiator frog (*Boana boans*). The frog got away, all the better as it may have been a meal too big to handle for the snake. If it had succeeded in biting the frog, the fight would have been far from over. This species of frog has been observed to escape from the jaws of snakes on at least two occasions, employing multiple defensive techniques such as emitting distress calls, jumping and lung inflation, making it harder for small snakes to hold on to them. Excellent survival strategies" (Fig. 7).

## Conclusions

Thank you to everyone who submitted an entry, we received many fantastic photos making the judging a wonderful experience for all editors involved. We hope that our readers enjoy viewing these images and discovering the stories behind them and join us in looking forward to another visual feast next year.
