# Correction to: Inaugural *BMC Ecology and Evolution* image competition: the winning images

**DOI:** 10.1186/s12862-021-01891-w

**Published:** 2021-09-09

**Authors:** Jennifer L. Harman, Alison L. Cuff, Josef Settele, Luke M. Jacobus, David A. Liberles, Arne Traulsen

**Affiliations:** 1 BMC, London, UK; 2 BMC, Berlin, Germany; 3 Helmholtz-Centre for Environmental Research – UFZ, Leipzig, Germany; 4 Indiana University-Purdue University Columbus (IUPUC), Columbus, IN USA; 5 Max-Planck Institute for Evolutionary Biology, Schleswig-Holstein, Germany; 6 Temple University, Philadelphia, PA USA

## Correction to: BMC Ecol Evo (2021) 21:157 https://doi.org/10.1186/s12862-021-01886-7

Following the publication of the original article [1], we were notified that:

1. The description of Fig. 2 was incorrect:
  This image shows "an amphipod crustacean of the species* E. verrucosus* densely covered with an overgrown colony of parasitic ciliates. Ciliates living on weakened crustaceans are capable of forming vast colonies resembling a "fur coat"
  Should read:
  This image shows "an amphipod crustacean of the species* E. verrucosus* densely covered with an overgrown colony of parasitic ciliates and unknown oomycetes or fungi. These organisms on weakened crustaceans are capable of forming vast colonies resembling a "fur coat"
2. The caption of Fig. 2 was changed from “*Eulimnogammarus verrucosus*, a species of crustacean endemic to the UNESCO World Heritage Site Lake Baikal, suffering from a parasitic ciliate infection. Attribution: Kseniya Vereshchagina” to “*Eulimnogammarus verrucosus*, a species of crustacean endemic to the UNESCO World Heritage Site Lake Baikal, suffering from a parasitic ciliate **and unknown oomycete (water mold) or fungi** infection. Attribution: Kseniya Vereshchagina”.
3. The affiliations of the 3rd and 4th authors had been swapped by mistake.

The original article has been corrected.
